# Mesenchymal Stem Cell Secretome for Cardiac Regeneration: Opportunity for Cell-Free Therapy

**DOI:** 10.3390/ijms27010209

**Published:** 2025-12-24

**Authors:** Paulina Piotrowska, Honorata Kraskiewicz, Aleksandra Klimczak

**Affiliations:** Laboratory of Biology of Stem and Neoplastic Cells, Hirszfeld Institute of Immunology and Experimental Therapy, Polish Academy of Sciences, R. Weigla 12, 53-114 Wroclaw, Poland; paulina.piotrowska@hirszfeld.pl (P.P.); honorata.kraskiewicz@hirszfeld.pl (H.K.)

**Keywords:** mesenchymal stem/stromal cells (MSCs), MSCs secretome, MSCs exosomes, myocardial infarction (MI), cell-free therapy

## Abstract

Cell-free therapy is gaining increasing interest among researchers as an alternative to mesenchymal stem/stromal cell (MSC) therapy. Since the therapeutic effects of MSCs rely predominantly on their paracrine activity, the use of their secretome as a therapeutic agent in broadly defined regenerative medicine, including cardiac regeneration, appears to be a rational approach. In this review, we discuss recent studies that employed secretomes derived from various types of MSCs in cardiomyocyte regeneration following myocardial infarction (MI). Special attention is given to the protein components of the secretome, which may drive tissue repair, and methods of priming the MSC to achieve secretome composition tailored for heart regeneration. Finally, we summarize recent preclinical findings on the effects of MSC secretomes on cardiomyocyte regeneration.

## 1. Introduction

### 1.1. Burden, Risk Factors, and Limitations of Current Treatments

According to the World Health Organization (WHO), 17.9 million people die annually of cardiovascular diseases (CVDs) which makes CVDs the main cause (approximately 32%) of death worldwide. CVDs is a general term for a group of conditions affecting heart and blood vessels including myocardial infarction, coronary heart disease, rheumatic heart disease, cerebral vascular disease, and other disorders [1]. Acute myocardial infarction (AMI) or myocardial infarction (MI), mostly named as a heart attack, is a serious and potentially life-threatening condition usually caused by the reduction or blockage of coronary artery blood flow, which leads to myocardial injury or necrosis. It leads to heart cells death and permanent damage to the heart muscle. The blood flow is generally limited by a blood clot in the coronary artery, and therefore oxygen and nutrients cannot be delivered to the heart muscle cells. Not only a decrease in the quantity of oxygen and other blood supplies is responsible for AMI. It is known that an increased amount of blood supply, caused by increased heart rate, may also lead to myocardial damage [2,3,4,5,6]. In addition to these physiological factors, there are several risk factors for myocardial infarction, include high blood pressure, high cholesterol, obesity, diabetes, smoking, and a family history of heart disease. Additionally, a sedentary lifestyle and poor diet also increase the risk of developing an MI [7].

Given the multiple risk factors for MI, preventive strategies are essential and may include anticoagulant medication, lifestyle changes, and procedures such as angioplasty, coronary artery bypass surgery, or implantation of a stent [8].

While prevention is essential, addressing damage after a heart attack is far more challenging. The most difficult aspect is the regenerative treatment of damaged tissue, because the current gold standard treatment for myocardial infarction focusses on revascularization to restore blood flow to the affected heart muscle, but it does not address the problem of cardiomyocyte loss. For the reason of the irreparable loss of cardiomyocytes and the accelerating aggravation of cardiac function, patients will have a reduced quality of life and increased risk of heart failure. A decrease in cardiomyocytes causes the remodelling of healthy tissue near the MI area. This remodelling consists of changes in the shape and size of the ventricles which can lead to the apoptosis or necrosis of cardiomyocytes and promote interstitial fibrosis [9,10,11,12].

### 1.2. Cardiac Progenitor Cells (CPCs) and Cardiac-Derived Stromal Cells (CSCs)

Given the limited regenerative capacity of the adult heart, research has increasingly focused on the potential of cardiac progenitor cells (CPCs) and cardiac-derived stromal cells (CSCs) to restore damaged myocardium. CPCs found in the human myocardium are characterized by self-renewal, clonogenic, and multipotent properties. In the steady-state conditions, resident progenitor cells are able to differentiate into cardiomyocytes and coronary vessels to maintain tissue homeostasis [13]. Resident CPCs are found in the atria, ventricles, epicardium, and pericardium and can be activated following an injury. Current research is focused on the characterization of endogenous CSCs which exhibit superior therapeutic potential due to their innate abilities to differentiate into cardiac cells, especially cardiomyocytes. The CPCs comprise the heterogeneous population of cells classified according to their biologic properties and surface markers for the following: side population cells (SPC), c-kit+ (CD117+), stem cell antigen-1 (Sca-1+), Isl-1+, cardiac atrial appendage stromal cells (CASCs), cardiac adipose-derived stromal cells (CADSCs), and cardiospheres [13,14,15]. Most of the CPCs are involved in cell growth and the proliferation and differentiation into cardiac cells.

Cardiac side population cells (cSPCs) are tissue-specific hematopoietic cells identified by the expression of VE-cadherin, CD31, CD34, and Sca-1 with the ability to differentiate into a diversity of cells responsible for cardiac function such as cardiomyocytes, endothelial cells, fibroblasts, and smooth muscle cells. C-kit is a tyrosine kinase receptor for the stem cell factor. Population of CSCs expressing c-kit+ are characterized by clonogenic/self-renewing properties that support the post-infarction healing process by reducing hypertrophy and fibrosis, thereby improving cardiac function. CSCs c-kit+ are able to differentiate into cardiomyocytes, endothelial cells, and smooth muscle cells, necessary for cardiac regeneration [13,14]. Sca-1+ cells play a crucial role in inhibiting cardiomyocyte apoptosis, post-infarction healing, and inducing stromal/progenitor cell differentiation into cardiomyocytes. However, CSCs Sca-1+ play a role in the healing process mainly due to angiogenesis facilitating blood flow restoration. Isl-1+ cells are considered as true cardiomyocyte progenitors of mesodermal origin, emerging during embryogenesis and contributing to the formation of cardiomyocytes, endothelial cells, and smooth muscle cells during heart development and cardiac neural crest cells [13,14].

CASCs are located in the atrial appendages and are phenotypically unique from other CSCs. CASCs secrete aldehyde dehydrogenase to protect against oxidative stress and secrete angiogenic factors including vascular endothelial growth factor (VEGF), endothelin-1 (ET-1), making them key candidates for regenerative therapies [14]. Another population of CPCs are CADSCs which are a specific population of progenitor cells isolated from the adipose tissue surrounding the heart, particularly the epicardial fat. These cells exhibit a mesenchymal stem/stromal cell (MSC)-like phenotype such as CD105, CD44, CD90, and CD29, and cardiac-specific biomarkers (MEF2C, NKX2-5, TBX5, IRX4). CADSCs secrete variety of factors that protect myocardial cells from apoptosis, inflammation, and fibrosis, and are able to differentiate into diverse cardiovascular cell types including cardiomyocytes [14].

Cardiosphere-derived cells (CDCs) comprise a heterogeneous cluster of CSCs expressing cardiac-specific factors (GATA4, MEF2C, and MKX2-5), surrounded by stromal cells. The regenerative potential of CDC is accomplished by paracrine activities promoting angiogenesis and apoptosis reduction [14].

Unfortunately, despite the abundant representation of myocardial progenitor cells in different compartments of the heart, they are unable to repair the extensive cardiac damage caused by MI. Therefore, alternative tissue sources of obtaining stem/progenitor cells capable of undertaking regenerative processes in the ischemic heart are widely studied.

### 1.3. Mesenchymal Stromal/Stem Cells (MSCs) in Cardiac Regeneration

Given the limitations of current therapies and the progressive remodelling that follows cardiomyocyte loss, there is a growing need for innovative regenerative approaches. In this context, mesenchymal stromal/stem cells (MSCs) have emerged as a promising approach for restoring heart muscle after a myocardial infarction. MSCs are non-hematopoietic, multipotent cells which are located in almost every organ and tissue. The International Society for Cellular Therapy established three biologic characteristics which must be met by MSC which are plastic adherence, the presence of CD73, CD90, and CD105 antigens, and the absence of CD34 and CD45 which are characteristic of hematopoietic cells. MSCs should also be able to differentiate into chondrocytes, adipocytes, and osteocytes [13,16,17,18,19]. Numerous studies have reported that MSCs possess a wide range of properties such as immunomodulatory, anti-fibrotic, proangiogenic, and anti-apoptotic effects. However, the beneficial properties of MSCs are limited by their paracrine action so the regenerative effect is correlated with the accuracy and quantity of transplanted cells [20,21,22,23]. Since the beginning of the 21st century, many clinical trials have been conducted using MSCs for the regeneration of the heart muscle. However, the improvement of cardiac function has not been satisfactory, since the reported benefits usually fall within the range of only 3% to 15% [24,25]. It has been shown that most transplanted MSCs die several days or weeks after transplantation [26]. Currently, it is known that MSCs do not differentiate into cardiomyocytes in the infarcted area; rather, they stimulate regeneration by their paracrine effects [27]. Therefore, using a conditioned medium from MSCs, which contains a variety of biologically active components, is a very interesting and rapidly developing approach in myocardial regeneration. It has been shown that regeneration through the MSC therapy relies on the activity of numerous cytokines and growth factors. These bioactive molecules orchestrate complex processes that promote cardiomyocyte proliferation and tissue repair. Among them, interleukin 6 (IL-6) plays a key role by influencing the proliferation and differentiation by the activation of the STAT3 signalling pathway in macrophages that play a major role in the proliferation of myoblasts in cardiac regeneration [25,28,29]. However, it should be emphasized that IL-6 is a pleiotropic cytokine, capable of both supporting repair processes and aggravating damage, especially by maintaining inflammation in chronic conditions [30]. Another important factor is a transforming growth factor β (TGF-β) which contributes to heart muscle regeneration by inducing myogenesis and suppressing inflammation [31]. Vascular endothelial growth factor (VEGF) plays an important role after cardiac ischemia as it regulates angiogenesis [25]. Several literature reports suggest that the activity of fibroblast growth factor (FGF) and VEGF is correlated. It is because the angiogenic activity of FGF is at least in part dependent on the presence of VEGF, as FGF has been shown to stimulate VEGF expression in endothelial cells. At the same time, VEGF-induced tubulogenesis requires the activation of FGF signalling. Moreover, in a murine model, an increase in neovascularization was associated with the high local expression of VEGF and FGF, implying that these factors play indispensable roles in postischemic angiogenesis [25,32].

In the present review, we discuss the literature regarding the application of various sources of MSC secretomes for cardiac regeneration, focusing on bioactive proteins secreted by MSCs and cell priming approaches which are being used in order to increase MSC secretory potential. We will also discuss the advantages of cell-free therapy, followed by a summary of recent research models and clinical trials carried out in this area.

## 2. MSC Secretome

The majority of mammalian cells can produce and secrete a mixture of biological active factors defined as cell secretomes. Especially, MSCs are highly active in this phenomenon. In the literature, the term “secretome” can be replaced by words such as “conditioned medium” or “supernatant”. Due to its multi-level action, in recent years, the MSC secretome has been widely studied in terms of its composition, biological activity, and potential therapy. The best known and most frequently studied are the regenerative properties such as the stimulation of differentiation and proliferation of the target cells, angiogenesis promotion, immunomodulation, and anti-apoptotic and anti-microbial activities [18].

### 2.1. MSC Secretome Composition

**Figure 1:**
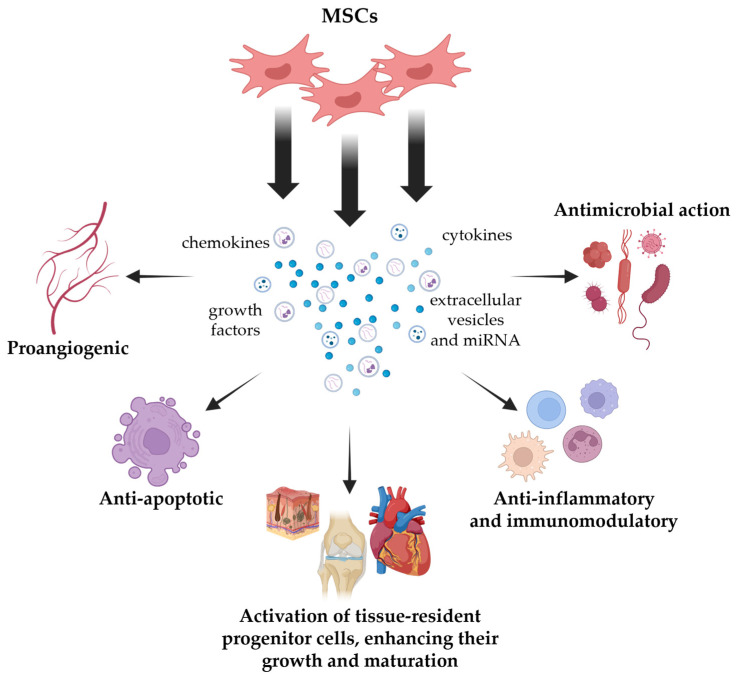
Composition and activity of the MSC secretome. Created in BioRender. Piotrowska, P. (2025) https://BioRender.com/77qcqbe.

The composition of the MSC secretome is very rich and consists of proteins, peptides, lipids, and nucleic acids. These molecules exhibit a wide range of biological activities and exist in both soluble and vascular form, as illustrated in Figure 1. Vascular fraction defined as extracellular vesicles (EVs) is further categorized based on the size of the EVs. The smallest are exosomes that range in size from 30 to 200 nm and originate from the endocytic pathway. Subsequently, there are microvesicles (MVs) and apoptotic bodies, with an average size 200 to 1000 nm and >1000 nm, respectively, and both of which originate from the plasma membrane [33].

The most extensively studied fraction of the MSC secretome is the protein/peptide component. Proteomic analysis of the secretome of the MSC reviles more than 1000 different proteins [34]. The specific protein content, both quantitative and qualitative, can also be analyzed by dedicated ELISA or Multiplex ELISA methods, which enable detection from single protein to dozen protein targets. Interestingly, qualitative and semi-quantitative protein analysis of the secretome can be performed using dedicated Membrane-Based Antibody Arrays, which allow the simultaneous assessment of several dozen proteins. An example of a secretome Antibody Array is presented in our previous research [35], where the production of 60 cytokines by four human adipose tissue-derived mesenchymal stem cell (HATMSC) lines was analyzed in our laboratory. However, it should be noted that the beneficial effects of MSCs are not solely mediated by secreted proteins. Studies have shown that MSCs from different sources can exert therapeutic effects through the release of bioactive lipids, such as lipoxins or prostaglandin, which play key roles in inflammatory regulation [36,37,38]. In addition, non-protein molecules, including nucleic acids such as mRNA, miRNA, and other non-coding RNAs also contribute significantly to their biological activity.

It is known that MSCs release a wide panel of cytokines and growth factors including IL-6, TGF-β, tumour necrosis factor α (TNF-α), VEGF, angiopoietin 1 (Ang-1), fibroblast growth factor 2 (FGF-2), and many others [25,39]. One of the research interests of our research group is the investigation of the MSC secretome for its potential use in regenerative medicine. We previously characterized the secretome of the HATMSC line derived from early-passage cells following isolation from the donor [40]. The secretome of adipose tissue-derived MSCs (ATMSCs), characterized through an extensive evaluation of primary cell cultures, shows a rich and consistent repertoire of cytokines and growth factors. Notably, six key cytokines—interleukin 8 (IL-8), matrix metalloproteinase-3 (MMP3), monocyte chemoattractant protein-1 (MCP1), growth-regulated protein (GRO), TGF-β, and tissue inhibitor of metalloproteinase (TIMP1)—were consistently detected across all ATMSC samples, forming a core set of signalling molecules. Additionally, several other cytokines, including Angiogenin, TIMP2, Angiopoietin 2, interferon-gamma-induced protein 10 (IP-10), and urokinase-type plasminogen activator receptor (uPAR), were frequently detected, further contributing to the complexity of the ATMSC secretome. The ATMSC secretome has a significant role in tissue regeneration, as evidenced by the presence of many biological factors crucial for tissue repair. Among the 43 human angiogenesis-related cytokines analyzed, 12 were consistently expressed at levels ≥ 5% across all ATMSC conditioned media, emphasizing the angiogenic potential of the secretome. Pro-angiogenic cytokines, such as angiogenin, GRO, IL-6, IL-8, and VEGF, were found abundantly in all ATMSC secretomes, suggesting a potent pro-regenerative influence. Furthermore, regulatory molecules like IGF-1, MCP-1, MMP-1, TIMP-1, and TIMP-2 were consistently present, further highlighting the multifaceted nature of the secretome. In another study, it was shown that the secretome profiles from MSCs differ significantly in terms of protein content depending on MSCs source, including the placenta (PL-MSCs), ADTMSCs, BM-MSCs, and WJ-MSCs. Proteome profiling analysis showed that the diversity and number of secreted proteins is significantly higher in MSCs derived from perinatal tissues (PL and WJ) compared to MSCs derived from adults (AD and BM). A total of 596 human proteins were identified, with the highest number found in PL-MSCs (511) and WJ-MSCs (440), while ADTMSCs (265) and BM-MSCs (253) showed less complexity in their composition. Furthermore, perinatal MSCs (PL-MSCs and WJ-MSCs) showed greater similarity in their secretome profile to each other than to adult MSCs, suggesting a biological link between these sources. However, it should be emphasized that despite these clear differences in proteome profile, functional analyses revealed that secretomes from different sources share similar overall functional characteristics, such as promoting cell migration and negatively regulating programmed cell death (anti-apoptotic effect) [41].

An important component of the MSC secretome is the microvesicles which play a crucial role in intercellular communication by carrying bioactive molecules, such as microRNAs (miRNAs), messenger RNAs (mRNAs), proteins, lipids, and other signalling molecules. In our studies on MSC, we also examined the MSC secretome for the presence of microvesicles and their content [42]. The study assessed the surface markers of HATMSC2 cells and their MVs. HATMSC2 cells expressed MSC markers CD73, CD90, and CD105, along with human leukocyte antigen (HLA) ABC antigen, while being negative for HLA DR antigen and the leukocyte marker CD45. Notably, HATMSC2-MVs also displayed these MSC markers (CD73, CD90, CD105, and HLA ABC) without expressing HLA DR or CD45, indicating that these microvesicles carry characteristics typical of parental MSCs. Furthermore, the studies confirmed the presence of specific microRNAs in the HATMSC2-MVs [42,43,44]. In turn, proteomic analysis of EVs derived from UC-MSCs revealed a complex and stable protein profile, crucial for their biological functions and therapeutic potential. A total of 807 unique proteins were identified, of which 676 constituted the core proteome, common to different donors and passages, indicating the high stability of the EV composition. The compatibility of 64 of these proteins with the most common EV markers in the ExoCarta database confirms their authenticity as components of extracellular vesicles. Functional analysis showed that the identified proteins, 92.9% of which were hydrophilic, are strongly involved in molecular and protein metabolism, material transport, and signal transduction (e.g., receptor and nucleotide binding) [45].

In another study on lipidomic analyses, samples of MSC-derived conditioned medium (MSC-CM) were analyzed, and the results show a panel of endocannabinoids and eicosanoids known to be involved in inflammation, as lipids might also play important roles in immune regulation. Seven lipid molecules, for example, arachidonoyl acid (AA), eicosapentaenoyl acid (EPA), docosahexaenoic acid (DHA), prostaglandin-E2 (PGE2), prostaglandin-F2α (PGF2α), N-palmitoylethanolamide (PEA), and N-stearoylethanolamide (SEA), were reliably quantified by UHPLC–MS/MS analysis in all conditioned medium (CM) samples [37]. In summary, the ATMSC secretome emerges as a complex and dynamic milieu of cytokines and growth factors, showcasing its potential as a powerful mediator of angiogenesis and tissue regeneration. The consistent presence of key factors across diverse MSC samples underlines the reliability and potency of the MSC secretome, making it a promising candidate for therapeutic applications in regenerative medicine, including cardiac regeneration.

### 2.2. Priming Approaches to Tailor MSC Secretome Composition

MSC priming has emerged as a promising strategy to enhance the therapeutic potential of MSC-based treatments. Research has shown that MSCs can be regulated by many external factors, each of which can distinctly influence the composition and biological activity of their secretome. By exposing MSCs to specific environmental cues, such as hypoxia, inflammatory cytokines, pharmacological agents, or biophysical stimuli, it is possible to shift their paracrine profile toward enhanced pro-angiogenic, cardioprotective, immunomodulatory, or anti-fibrotic functions.

The most frequently used priming method is hypoxia, which mimics the ischemic environment and upregulates important signalling molecules in the cardiovascular system such as VEGF, Hepatocyte growth factor (HGF), and insulin-like growth factor-1 (IGF-1) [46]. Thus, it has been shown that exosomes from hypoxia-preconditioned bone marrow MSCs reduce apoptosis in cardiomyocytes [47] or that small EVs from hypoxia-treated MSCs can improve cardiac function after MI, partly via a miRNA-mediated pro-angiogenic mechanism [48].

Another strategy involves priming MSCs with pro-inflammatory cytokines such as TNF-α, interferon gamma (IFN-γ), or IL-1β, which mimics inflammatory niches in injured cardiac tissue, and consequently enhances their reparative and immunomodulatory functions. Recently, it was shown that the priming of Wharton’s jelly-derived MSCs (WJ-MSCs) by IL-1β, TNF-α, and IL-17 enhanced the anti-inflammatory secretions and the primed secretome was able to polarize macrophages from the inflammatory M1 phenotype to the anti-inflammatory M2 phenotype [49]. Several research studies demonstrated that priming MSCs with pro-inflammatory cytokines shifts their secretome toward an immunomodulatory and anti-fibrotic profile, including the increased production of indoleamine 2,3-dioxygenase (IDO), PGE_2_, and interleukin-10 (IL-10) [50,51], all of which are important for cardiac remodelling after myocardial infarction [52]. There are also some reports demonstrating that the immunomodulatory priming of MSCs enhances their therapeutic efficacy, particularly improving outcomes in cardiac injury models. For example, it was shown that exosomes derived from IFN-γ-primed MSCs exert stronger cardioprotective effects in a myocardial infarction model, including reduced tissue damage and improved cardiac function. The enhanced effect is linked to changes in the paracrine cargo induced by inflammatory priming. Interestingly, several studies have also employed lipopolysaccharides (LPS) as a preconditioning stimulus for MSCs and reported measurable therapeutic benefits in cardiac injury models [53]; however, in our view, despite these effects, LPS-based priming is unlikely to be clinically applicable due to safety concerns and its incompatibility with human therapeutic use.

It is also worth noting that MSCs for cardiovascular therapy can be primed using various chemical agents such as deferoxamine (DFO) or FGF-2 or TGF-β inhibitors [54]. Although such pharmacological priming has been applied predominantly to enhance the quality, survival, and reparative potential of MSCs for cell-based transplantation, emerging studies indicate that, for example, exosomes derived from atorvastatin-pretreated MSCs improved cardiac repair in rodent AMI models via pro-angiogenic and immunomodulatory mechanisms [55,56]. Therefore, secretomes derived from chemically primed MSCs may likewise exhibit improved therapeutic properties and could be considered for future cell-free applications.

Other promising strategies for MSC priming, such as biophysical stimulation and genetic manipulation, have also been explored. In the specific context of cardiac regeneration, studies using these approaches to generate therapeutic secretomes remain relatively limited. However, in a rat model of myocardial infarction it has been shown that exosomes derived from human umbilical cord MSCs (UC-MSCs) that overexpress hypoxia-inducible factor 1 (HIF-1α) significantly reduced cardiomyocyte apoptosis, enhanced angiogenesis, reduced scar size, and improved cardiac function [57]. Similarly, it was reported that MSCs engineered to overexpress CXCR4 by lentiviral transduction secrete exosomes that, when applied to a rat MI model, improved angiogenesis, reduced infarct size, and enhanced cardiac remodelling [58].

Studies conducted by our research group also confirmed that priming significantly influences the composition of the MSC secretome. We established an immortalized MSC line (HATMSC1) from human adipose tissue that produces a rich and biologically diverse secretome, notably increasing pro-angiogenic factors (e.g., VEGF, IL-8, angiogenin) under hypoxic conditions [35]. Moreover, the exposure of these cells to inflammatory stimuli, such as TNF-α or IFN-γ, further enhanced the secretion of cytokines and growth factors [59], highlighting the potential of controlled priming to generate a potent, cell-free therapeutic product for regenerative medicine and immunotherapy. Understanding and optimizing these priming strategies is crucial for improving the therapeutic outcomes of MSC- based treatments in various diseases, particularly those involving inflammation and tissue regeneration.

### 2.3. Methods of Preparation of MSC Secretomes

Based on the studies cited in this review (Table 1), it is evident that the process of MSC secretome production is highly variable. The preparation of the secretome differs not only in the method used to stimulate MSCs to produce the conditioned medium, but also in the production process itself and the origin of the MSCs. Across multiple studies, an MSC secretome was produced using well-characterized MSCs derived from tonsils [60], adipose tissue [35,40,42,43,44], bone marrow [61,62,63], or umbilical cord [64,65]. These cells were expanded under controlled conditions that allowed the accumulation of their paracrine factors in the surrounding medium.

Cells maintained at low passage numbers better preserved their paracrine activity. A high level of confluence ensures stable secretory profiles. After the culture medium is replaced, MSCs are allowed to condition it for a defined period (12, 24, 48, or 72 h), enabling the accumulation of bioactive paracrine factors. The MSC handling protocol—maintenance in standard medium or brief exposure to defined cytokine stimulation prior to secretome collection—depended on the experimental objective, such as therapeutic application or immunomodulation. In each case, the conditioned medium was collected after a defined incubation period, cleared out of cellular debris, and subsequently concentrated to enrich its bioactive components and thereby increase biological potency. Interestingly, it has been proven that the secretome profile of each MSC population (adipose tissue, bone marrow, placenta, and Wharton’s jelly) (Table 1) has distinct characteristics depending on the cell source. It was found that MSCs derived from perinatal tissues (placenta and Wharton’s jelly) had more diverse compositions of selected proteins, which may suggest their greater therapeutic potential [41]. Although MSCs from different sources demonstrated similar immunosuppressive activity and differentiation capacity, UCB-MSCs (from umbilical cord blood) showed stronger anti-inflammatory effects, for example, in LPS-induced inflammation. It was established that this beneficial effect was partly related to the greater secretion of the paracrine factor Angiopoietin-1 (Ang-1) by cells derived from umbilical cord blood [66]. The concentrations of many bioactive factors in the medium conditioned by UC-MSCs were ten to one hundred thousand times higher compared to a medium conditioned by bone marrow-derived MSCs (BM-MSCs). This suggests that cell-free therapy products derived from the umbilical cord may have a stronger therapeutic effect [67]. In summary, although all MSC secretions exhibit general regenerative and immunomodulatory effects, their potential and specific therapeutic effects may vary depending on a variety of factors, such as the cell source (tissue of origin, donor age), cultivation conditions (culture medium, duration of culture, confluence), and priming methods (hypoxia, immunostimulation, chemical or genetic treatments). This methodological diversity makes it difficult to compare results across studies and highlights the need for standardized secretome preparation protocols for clinical applications.

**Table 1:** Overview of breeding conditions and harvesting techniques of MSC secretome.

	Parameter	Cell Type	Passage/ Confluence	Base Medium	CM Induction Medium	Induction Time	Pre- Treatment	Concentration Method	Concentration Factor	Cut-Off (kDa)
Ref.										
[60]		Tonsil-derived MSCs	P7–9, 80%	Low-glucose DMEM	Low- glucose DMEM (serum-free implied)	48 h	None specified	Amicon Ultra (centrifugal)	20-fold	Not specified
[68]		hMSCs (ATCC)	Confluence	α- MEM	Serum-free	48 h	None specified	Amicon Ultra-15 (centrifugal)	Not specified	100 kDa
[69]		MSCs	90%	DMEM	Serum-free	12 h	20 ng/mL IFN-γ TNF-α	Ultrafiltration (membrane)	Not specified	3 kDa
[70]		WJ-MSCs	P4, 80%	DMEM	hPL-free DMEM	24, 48, 72, 96 h	None specified	Ultrafiltration	Not specified	Not specified
[64]		UC-MSCs	80–90%	L-DMEM	Serum-free	48 h	Akt Transfection	Ultrafiltration (membrane)	Not specified	100 kDa

## 3. Advantages and Challenges of MSC-Derived Cell-Free Therapy

MSCs, the prominent stem cells in the field of cell-based therapy, have been used in clinical trials for cardiovascular diseases for more than 20 years [71]. Despite the huge amount of research in this field, the results of clinical studies are still not satisfactory. The efficacy of stem cell-based myocardial infarction therapy remains limited by several challenges, including poor cell survival, low retention rates, poor integration, and limited functional outcomes [72]. Therefore, MSC-based therapy is still not considered to be the standard of care at the clinic and is still debatable. However, preclinical studies have demonstrated that MSC-derived secretomes or exosomes can reproduce therapeutic effects similar to, or even exceeding, those of MSC transplantation. For example, MSC exosomes displayed very similar miRNA expression profiles and provided superior cardiac repair compared to MSCs in a rat MI model [61]. In another study, authors compared adipose-derived MSCs with the MSC-conditioned medium in a mouse MI model and found that both treatments reduced infarct size, decreased cardiomyocyte apoptosis, and improved cardiac function, supporting that paracrine secretions are the main mediators of MSCs’ benefits [73]. It was also shown that the conditioned medium from HIF-1α-enhanced MSCs reduced fibrosis, apoptosis, and infarct size in rat MI, showing that the modulation of MSCs alters secretome potency and that the CM itself has therapeutic activity [74]. The above studies suggest that the development of cell-free MSC-derived secretome therapies represents a strong and promising alternative to conventional cell-based approaches for myocardial repair. It is also worth emphasizing that the MSC secretome may not only serve as an alternative to cell-based therapy but may even represent a superior therapeutic option. Several factors deserve to be analyzed in this context. First of all, there is a safety advantage to the usage of secretomes over cell transplantation. Following the administration of the secretome, no risk of uncontrolled cell proliferation or tumorigenicity will occur. Secondly, in the case of the secretome, proper standardization can be more easily maintained, as it can be produced in a controlled and reproducible manner. Moreover, from a practical perspective the secretome, unlike living cells, can be stored, and its lyophilization can greatly extend its shelf life, making it very attractive for the pharmaceutical industry. It is important to add that the secretome can be concentrated and given at a controlled and defined dose, suitable for repeated administration, which is essential for chronic heart disease or remodelling phases post-MI. Interestingly, the secretome can also be easily combined with more sophisticated drug delivery systems, such as injectable hydrogels, nanoparticles, cardiac patches, or scaffolds, enabling sustained therapeutic effects. Finally, in the case of the clinical use of secretomes, the regulatory alignment would be easier while the cost of production lower compared to living cells. Finally, we would like to discuss one more issue, namely, the use of secretomes produced not by the patient’s primary cells, but by an immortalized MSC line. In our research work, we have successfully established a human MSC line of adipose tissue origin (HATMSC) that produces a cocktail of growth factors and cytokines involved in tissue regeneration [40]. The use of established stem cell lines for secretome production provides additional advantages, above all the minimal invasiveness, since it reduces a need for harvesting MSCs from patients. The proposed solution also avoids variability of the secretome resulting from donor age and disease status. Finally, the secretome produced by the established cell line would be much more widely available.

Nevertheless, to provide a comprehensive and balanced discussion of the topic, it is important to acknowledge that several significant limitations and standardization challenges continue to hinder the translation of this therapy into clinical practice. The composition of the MSC-derived secretome is highly heterogeneous and this may affect its biological activity and reproducibility across different batches and laboratories. Another significant problem involves a lack of standardized isolation protocols and unclear regulatory guidelines for good manufacture practice GMP production [75]. An important factor, especially in cardiac muscle regeneration, is the method of drug administration. In comparison to direct administration, after intravenous injection of EVs/exosomes for the treatment of cardiac injury, only a very small fraction actually reaches the myocardium [76]. Therefore, to achieve satisfactory treatment outcomes, the administration of the secretome is comparably challenging as in the case of cell therapy.

## 4. Recent Preclinical Findings in Cardiac Regeneration

**Figure 2:**
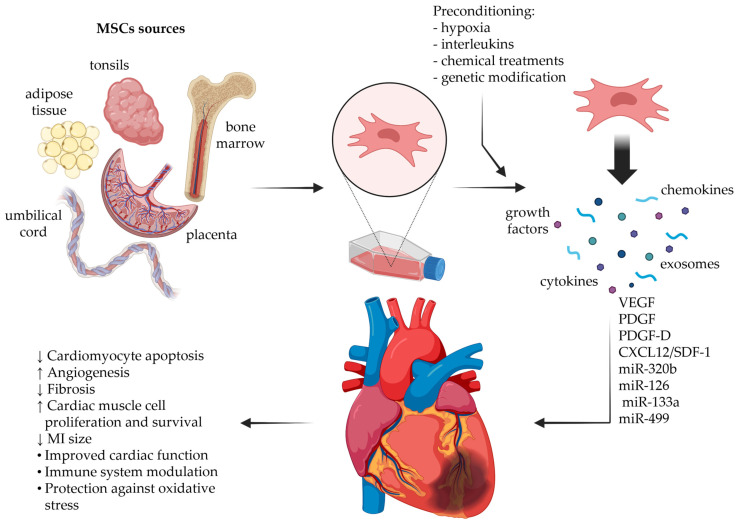
A schematic representation of MSC secretome preparation and its therapeutic impact on cardiomyocyte regeneration following myocardial infraction. Created in BioRender. Piotrowska, P. (2025) https://BioRender.com/ulnxutz.

Recent studies have increasingly emphasized the pivotal role of the MSC secretome in MI repair. While early research focused primarily on direct cell engraftment, accumulating evidence indicates that the therapeutic benefits of MSCs arise largely from their paracrine activity rather than from durable cell integration. Reports demonstrate that hypoxic cardiomyocytes treated with the MSC-derived secretome exhibit significantly increased expression of HIF-1α, RhoA, and IL-18. This suggests that MSC-secreted factors enhance cellular adaptation to hypoxia, activate RhoA-mediated structural and stress signalling pathways, and intensify inflammatory responses within cardiomyocytes [77,78,79]. Since the protective effect of the secretome has been demonstrated in an in vitro model, researchers worldwide have initiated in vivo studies. A summary of recent experimental findings in this area is presented in Table 2, and a schematic representation of the experimental design and treatment outcomes is shown in Figure 2. Research is primarily focused on determining whether the therapeutic effects of MSCs can be achieved by using their secretome, and in particular the exosomes instead of the cells themselves. Enriching exosomes through protein kinase B (Akt) overexpression was an interesting approach. Exosomes derived from Akt-modified human umbilical cord-derived MSCs (Akt-Exo) exhibited superior cardioprotective effects following MI, highlighting the functional importance of MSC paracrine signalling rather than direct cell engraftment. Importantly, Akt-Exo had a significant impact on the repair of MI in the rat model promoting angiogenesis. Proteomic analysis revealed that these exosomes contain elevated levels of PDGF-D, a key mediator responsible for angiogenic responses. Collectively, the data underscore that the targeted modification of the MSC secretome can substantially amplify its regenerative potential and represents a promising strategy for post-infarction cardiac repair [64]. These results are similar to those obtained by Alrefai and colleagues [63] which also demonstrate a significant improvement of cardiac function in the rat model of MI; however, in their settings human bone marrow-derived MSCs were used for secretome production. Moreover, the use of human-derived cells in different animal models is a very common practise, for example, human embryonic MSC line-derived exosomes were applied to a porcine model which resulted in a 30–40% reduction in infarct size. This reduction was observed in both early and late time points after MI, demonstrating a strong secretome-mediated cardioprotective effect. Exosome treatment also decreased infarct transmurality and limited wall thinning, indicating the attenuation of adverse remodelling. Pigs that received exosomes exhibited relative preservation of regional LV function, particularly fractional wall thickening, compared with controls. Although global LV function was less protected, this may relate to the increased fibrosis observed in the exosome-treated segments [80]. However, in the current research, the usage of human-derived MSCs to produce secretomes for animal applications is not the only approach. It is equally common to use MSCs derived from the same species to which the treatment is applied. It has been demonstrated that single injections of GATA-4-expressing mice-derived bone marrow MSC exosomes applied to mice after MI led to a significant and sustained improvement in cardiac function within 96 h after administration. The improvement in global systolic function was accompanied by significant histopathological changes in the damaged myocardium: an increase in the blood vessel density (angiogenesis), an increase in the number of endogenous stem cells, and a reduction in the number of apoptotic cardiomyocytes. Analysis of the differential expression of microRNA and protein candidates in exosomes confirmed the hypothesis that these packages of biologically active molecules act as the effectors responsible for inducing regeneration, modulating differentiation, and inhibiting cell death [62].

The route of secretome delivery to tissues also varies. The intravenous administration of the secretome is a commonly used method [76], as is treatment via hydrogels containing secretomes. In order to optimize delivery, sEVs were incorporated into an alginate hydrogel (sEVs-Gel). The study showed that this delivery system significantly increased the retention of sEVs in the heart tissue. This improved bioavailability of the secretome thus improved functional and biological outcomes. Early effects of this treatment included a reduction in cardiomyocyte apoptosis and the promotion of macrophage polarization towards reparative phenotypes. In the long term (after 4 weeks), sEVs-Gel therapy led to increased angiogenesis and improved cardiac parameters, including reduced infarct size and improved systolic function. This confirmed the hypothesis that the incorporation of sEVs into biomaterials is an effective strategy for sustainable and effective regenerative therapy [81].

To ensure the timeliness and relevance of the review, the PubMed and ClinicalTrials.gov database searches were strictly limited to publications from January 2017 to December 2025. This specific time allowed us to focus on the latest evidence and research trends while avoiding the inclusion of potentially outdated information. The use of a time filter was precisely included in the final search string, immediately after applying the keywords, ensuring completeness and consistency with the defined inclusion criteria. The following keywords were used: MSC secretome/exosomes cardiac regeneration/repair; MSC secretome (protein) composition; MSC myocardial infraction regeneration; MI regeneration/repair; MSC cardiomyocytes survival.

To conclude, the MSC secretome has been shown to modulate inflammation, promote angiogenesis, and enhance cardiomyocyte survival after MI. These findings have shifted the field toward cell-free regenerative strategies, positioning the MSC-derived secretome as a promising and potentially safer alternative to conventional stem cell transplantation in post-infarction cardiac regeneration.

**Table 2:** The effect of the MSC secretome on the cardiomyocyte regeneration process: evidence from in vitro and in vivo studies.

Cells/Animal	Form (Dose)	Types of MSCs	Secretome/ Exosomes	Result	Ref.
Cardiomyocytes isolated from rat hearts	30 ug/mL	MSC (human ATCC)	Exosomes	Improvement of cell viability and reduction in apoptosis; pyroptosis inhibition	[82]
Mouse and H9c2 cells	50 μL of CM (4 μg/mL	AT-MSC (human)	Secretome	Decreased apoptosis and fibrosis	[79]
Rat	400 μg	UC-MSC (human)	Secretome	Improved cardiac function, decreased apoptosis, increased angiogenesis	[78]
Rat	20 μg/20 μL	UC-MSC (human)	Exosomes	Improved cardiac function, reduced MI size, decreased inflammation	[64]
Rat	600 μL	BM-MSC (rat)	Exosomes	Improved cardiac function, reduced MI size	[61]
Mouse	20 μg/mL	BM-MSC (mouse)	Secretome	Improved cardiac function, decreased apoptosis, increased angiogenesis	[83]
Mouse	50 μg/30 μL	Cardiac- MSC (mouse)	Exosomes	Improved cardiac function, increased angiogenesis	[62]
Mouse	0.5 μg/μL	BM-MSC (mouse)	Exosomes	Decreased apoptosis	[84]
Mouse	50 μg/100 μL	BM-MSC (mouse)	Exosomes	Improved cardiac function, increased angiogenesis, decreased MI size, decreased inflammation	[85]
Mouse	600 μg/20 μL	BM-MSC (mouse)	Exosomes	Improved cardiac function, increased angiogenesis	[86]
Mouse	5 μg/25 μL	BM-MSC (mouse)	Exosomes	Improved cardiac function, decreased apoptosis	[87]
Rat	40 μg/300 μL	MSC (human ATCC)	Exosomes	Decreased MI size, apoptosis, and inflammation	[88]
Rat	50 μg/mL	UC- MSC (human Shycbio)	Exosomes	Improved cardiac function, decreased MI size and apoptosis, increased angiogenesis	[65]
Rat	80 μg/200 μL	BM-MSC (rat)	Extracellular vesicles	Improved cardiac function, decreased MI size, decreased inflammation	[81]
Rat	500 μL	BM-MSC (human)	Secretome	Decreased MI size, increased angiogenesis	[63]
Mouse	50 μg/25 μL	BM-MSC (mouse)	Exosomes	Decreased MI size and inflammation	[89]
Mouse	20 μg/30 μL	MSC (not specified)	Exosomes	Improved cardiac function, decreased MI size, increased angiogenesis	[90]
Mouse	10 μg/100 μL	BM-MSC (rat)	Extracellular vesicles	Improved cardiac function, decreased collagen volume	[91]
Pig	1000 μg	Embryo MSC (human cell line)	Exosomes	Reduced infarct size and protected regional cardiac wall function	[80]
Mouse, Rat, Pig	9 × 1010 particles/mL	MSC (not specified)	Exosomes	Improved cardiac function, decreased MI size	[92]

## 5. Conclusions and Further Directions

Clinical problems resulting from MI, which led to chronic heart failure due to the irreversible loss of cardiomyocytes, represent an urgent and unmet clinical need for effective myocardial regenerative therapies. Based on a detailed analysis of the recent literature, it can be concluded that the use of MSCs and their secretome has become well-established in regenerative medicine. The results shown are convincing, although areas requiring further refinement have been identified.

In the literature on the MSC secretome for cardiac regeneration after MI, most studies focus on extracellular vesicles, exosomes, or microvesicles rather than the whole secretome. In the context of the pharmaceuticalisation of therapy, the preferential use of exosomes and EVs over the whole secretome or CM stems from their advantages in quality control, safety, and stability. The advantages of EVs include the possibility of precise dosing and standardization through the quantitative assessment of vesicles, including well defined concentration, particle number, and size, which is crucial for clinical trials. EVs also provide selective therapy such as miRNA, proteins, and increased in vivo stability thanks to the protection of the cargo by the lipid membrane. There are also some disadvantages of using vesicles, mainly the complex and expensive isolation, which requires ultracentrifugation, filtration, or commercial kits. The whole secretome, on the other hand, is characterized by heterogeneity (including the risk of contamination with pro-inflammatory cytokines) and a lack of dosing precision, which hinders standardization. Although it is biologically more complete and may exhibit greater synergy in vivo, its complexity is a disadvantage in the pharmaceutical manufacturing process.

The key limitation of MSC secretome/macrovesicle therapy remains the inefficiency of therapeutic material delivery. Reports of improved cardiac function without a reduction in fibrosis may be attributed to the low targeting efficiency after intravenous administration [76]. Most EVs/secretomes are rapidly removed from the bloodstream and distributed to other organs, such as the liver or spleen, instead of concentrating in the myocardium. In turn, a local, intramuscular administration is invasive and carries the risk of heart damage or arrhythmia, which limits its clinical application. Finally, it is important to highlight that most studies on the MSC secretome or EVs for cardiac repair are in in vitro or animal models. Even though the preclinical evidence for the MSC secretome/EVs in cardiac repair is promising, we did not find any clinical trials explicitly using the MSC secretome (conditioned medium) or isolated exosomes/microvesicles for myocardial regeneration while searching the world’s largest clinical trials databases run by the US National Library of Medicine at the National Institutes of Health. Nevertheless, several clinical trials investigating the use of MSCs in the treatment of myocardial infarction have been registered. Using the search terms “Mesenchymal Stem Cells” and “myocardial infarction”, 32 registered clinical studies were identified in the same database. There could be several reasons for this, but mainly translational challenges from preclinical studies which include dosing, biodistribution, and delivery methods that are not yet fully optimized. Also, isolation and standardization methods are challenging and require further consideration. However, many clinical trials for myocardial regeneration currently focus on direct MSC therapy, which is already better studied in humans.

The above conclusions aim to summarize the main achievements and present aspects worth refining in the use of MSC-derived secretomes in myocardial regeneration after myocardial infarction.

## Data Availability

No new data were created or analyzed in this study. Data sharing is not applicable to this article.

## Abbreviations

The following abbreviations are used in this manuscript:

AA	Arachidonoyl acid
ACE	Angiotensin-converting enzyme
Akt	Protein kinase B
Akt- Exo	Akt-modified human umbilical cord-derived MSCs
AMI	Acute myocardial infarction
Ang-1	Angiopoietin 1
ATMSCs	Adipose tissue-derived MSCs
BM-MSCs	Bone marrow-derived MSCs
CASCs	Cardiac atrial appendage stromal cells
CADSCs	Cardiac adipose-derived stromal cells
CDC	Cardiosphere-derived cells
CM	Conditioned medium
CPCs	Cardiac progenitor cells
CSCs	Cardiac-derived stromal cells
cSPCs	Cardiac side population cells
CVDs	Cardiovascular diseases
DFO	Deferoxamine
DHA	Docosahexaenoic acid
DMEM	Dulbeco’s modified Eagle’s medium
ECG	Electrocardiogram
EPA	Eicosapentaenoyl acid
EVs	Extracellular vesicles
FGF	Fibroblast growth factor
FGF-2	Fibroblast growth factor 2
GMP	Good manufacture practice
GRO	Growth-regulated protein
HATMSCs	Human adipose tissue-derived mesenchymal stem cells
HGF	Hepatocyte growth factor
HIF-1α	Hypoxia-inducible factor 1
HLA	Human leukocyte antigen
HMSCs	Human-derived MSCs
hPL	Human platelet lysate
IDO	Indoleamine 2,3-dioxygenase
IFN-γ	Interferon gamma
IGF-1	Insulin-like growth factor-1
IL-10	Interleukin-10
IL-6	Interleukin-6
IL-8	Interleukin-8
IP-10	Interferon-gamma-induced protein 10
LPS	Lipopolysaccharides
MCP1	Monocyte chemoattractant protein-1
MI	Myocardial infarction
miRNAs	microRNAs
MMP3	Matrix metalloproteinase-3
mRNAs	Messenger RNAs
MSC-CM	MSC-derived conditioned medium
MSCs	Mesenchymal/stromal stem cells
MVs	Microvesicles
PEA	N-palmitoylethanolamide
PGE2	Prostaglandin-E2
PGF2α	Prostaglandin-F2α
Sca-1+	Stem cell antigen-1
SEA	N-stearoylethanolamide
sEVs-Ge	sEVs incorporated into an alginate hydrogel
SPCs	Side population cells
TGF-β	Transforming growth factor β
TIMP1	Tissue inhibitor of metalloproteinases
TNF-α	Tumour necrosis factor α
UC- MSCs	Umbilical cord MSCs
uPAR	Urokinase-type plasminogen activator receptor
VEGF	Vascular endothelial growth factor
WHO	World Health Organization
WJ-MSCs	Wharton’s jelly-derived MSCs
αMEM	Modified minimum essential medium

## References

[1] Roth G.A., Mensah G.A., Fuster V. (2020). The Global Burden of Cardiovascular Diseases and Risks: A Compass for Global Action. J. Am. Coll. Cardiol..

[2] Lindahl B., Mills N.L. (2023). A new clinical classification of acute myocardial infarction. Nat. Med..

[3] Shafei A.E., Ali M.A., Ghanem H.G., Shehata A.I., Abdelgawad A.A., Handal H.R., Talaat K.A., Ashaal A.E., El-Shal A.S. (2017). Mesenchymal stem cell therapy: A promising cell-based therapy for treatment of myocardial infarction. J. Gene Med..

[4] Thygesen K., Alpert J.S., White H.D., Jaffe A.S., Apple F.S., Galvani M., Katus H.A., Newby L.K., Ravkilde J., Joint ESC/ACCF/AHA/WHF Task Force for the Redefinition of Myocardial Infarction (2007). Universal definition of myocardial infarction. Circulation.

[5] Thygesen K., Alpert J.S., Jaffe A.S., Simoons M.L., Chaitman B.R., White H.D., Katus H.A., Lindahl B., Morrow D.A., Joint ESC/ACCF/AHA/WHF Task Force for the Universal Definition of Myocardial Infarction (2012). Third universal definition of myocardial infarction. Circulation.

[6] Saleh M., Ambrose J.A. (2018). Understanding myocardial infarction. F1000Res.

[7] Karbasiafshar C., Sellke F.W., Abid M.R. (2021). Mesenchymal stem cell-derived extracellular vesicles in the failing heart: Past, present, and future. Am. J. Physiol. Heart Circ. Physiol..

[8] Boateng S., Sanborn T. (2013). Acute myocardial infarction. Dis. Mon..

[9] Braunwald E., Bristow M.R. (2000). Congestive heart failure: Fifty years of progress. Circulation.

[10] Chien K.R. (1999). Stress pathways and heart failure. Cell.

[11] Hunter J.J., Chien K.R. (1999). Signaling pathways for cardiac hypertrophy and failure. N. Engl. J. Med..

[12] Engel F.B. (2005). Cardiomyocyte proliferation: A platform for mammalian cardiac repair. Cell Cycle.

[13] Klimczak A., Kozlowska U. (2016). Mesenchymal Stromal Cells and Tissue-Specific Progenitor Cells: Their Role in Tissue Homeostasis. Stem Cells Int..

[14] Inouye K., White G., Khan S., Luba J., Benharash P., Thankam F.G. (2025). Heart-derived endogenous stem cells. Mol. Biol. Rep..

[15] Bollini S., Smart N., Riley P.R. (2011). Resident cardiac progenitor cells: At the heart of regeneration. J. Mol. Cell Cardiol..

[16] Dominici M., Le Blanc K., Mueller I., Slaper-Cortenbach I., Marini F., Krause D., Deans R., Keating A., Prockop D., Horwitz E. (2006). Minimal criteria for defining multipotent mesenchymal stromal cells. The International Society for Cellular Therapy position statement. Cytotherapy.

[17] Brown C., McKee C., Bakshi S., Walker K., Hakman E., Halassy S., Svinarich D., Dodds R., Govind C.K., Chaudhry G.R. (2019). Mesenchymal stem cells: Cell therapy and regeneration potential. J. Tissue Eng. Regen. Med..

[18] Krawczenko A., Klimczak A. (2022). Adipose Tissue-Derived Mesenchymal Stem/Stromal Cells and Their Contribution to Angiogenic Processes in Tissue Regeneration. Int. J. Mol. Sci..

[19] Viswanathan S., Shi Y., Galipeau J., Krampera M., Leblanc K., Martin I., Nolta J., Phinney D.G., Sensebe L. (2019). Mesenchymal stem versus stromal cells: International Society for Cell & Gene Therapy (ISCT(R)) Mesenchymal Stromal Cell committee position statement on nomenclature. Cytotherapy.

[20] Van Linthout S., Stamm C., Schultheiss H.P., Tschope C. (2011). Mesenchymal stem cells and inflammatory cardiomyopathy: Cardiac homing and beyond. Cardiol. Res. Pract..

[21] Banerjee M.N., Bolli R., Hare J.M. (2018). Clinical Studies of Cell Therapy in Cardiovascular Medicine: Recent Developments and Future Directions. Circ. Res..

[22] Martinez-Falguera D., Iborra-Egea O., Galvez-Monton C. (2021). iPSC Therapy for Myocardial Infarction in Large Animal Models: Land of Hope and Dreams. Biomedicines.

[23] Csobonyeiova M., Beerova N., Klein M., Debreova-Cehakova M., Danisovic L. (2022). Cell-Based and Selected Cell-Free Therapies for Myocardial Infarction: How Do They Compare to the Current Treatment Options?. Int. J. Mol. Sci..

[24] Fuh E., Brinton T.J. (2009). Bone marrow stem cells for the treatment of ischemic heart disease: A clinical trial review. J. Cardiovasc. Transl. Res..

[25] Li N., Wang C., Jia L., Du J. (2014). Heart regeneration, stem cells, and cytokines. Regen. Med. Res..

[26] Mirotsou M., Zhang Z., Deb A., Zhang L., Gnecchi M., Noiseux N., Mu H., Pachori A., Dzau V. (2007). Secreted frizzled related protein 2 (Sfrp2) is the key Akt-mesenchymal stem cell-released paracrine factor mediating myocardial survival and repair. Proc. Natl. Acad. Sci. USA.

[27] Laflamme M.A., Murry C.E. (2011). Heart regeneration. Nature.

[28] Shao L., Shen Y., Ren C., Kobayashi S., Asahara T., Yang J. (2022). Inflammation in myocardial infarction: Roles of mesenchymal stem cells and their secretome. Cell Death Discov..

[29] Tang P., Ma S., Dong M., Wang J., Chai S., Liu T., Li J. (2018). Effect of interleukin-6 on myocardial regeneration in mice after cardiac injury. Biomed. Pharmacother..

[30] Katkenov N., Mukhatayev Z., Kozhakhmetov S., Sailybayeva A., Bekbossynova M., Kushugulova A. (2024). Systematic Review on the Role of IL-6 and IL-1beta in Cardiovascular Diseases. J. Cardiovasc. Dev. Dis..

[31] Abarbanell A.M., Coffey A.C., Fehrenbacher J.W., Beckman D.J., Herrmann J.L., Weil B., Meldrum D.R. (2009). Proinflammatory cytokine effects on mesenchymal stem cell therapy for the ischemic heart. Ann. Thorac. Surg..

[32] Murakami M., Simons M. (2008). Fibroblast growth factor regulation of neovascularization. Curr. Opin. Hematol..

[33] Doyle L.M., Wang M.Z. (2019). Overview of Extracellular Vesicles, Their Origin, Composition, Purpose, and Methods for Exosome Isolation and Analysis. Cells.

[34] Madonna R., Angelucci S., Di Giuseppe F., Doria V., Giricz Z., Gorbe A., Ferdinandy P., De Caterina R. (2019). Proteomic analysis of the secretome of adipose tissue-derived murine mesenchymal cells overexpressing telomerase and myocardin. J. Mol. Cell Cardiol..

[35] Kraskiewicz H., Paprocka M., Bielawska-Pohl A., Krawczenko A., Panek K., Kaczynska J., Szyposzynska A., Psurski M., Kuropka P., Klimczak A. (2020). Can supernatant from immortalized adipose tissue MSC replace cell therapy? An in vitro study in chronic wounds model. Stem Cell Res. Ther..

[36] Das U.N. (2020). Bioactive Lipids as Mediators of the Beneficial Action(s) of Mesenchymal Stem Cells in COVID-19. Aging Dis..

[37] Casati S., Giannasi C., Niada S., Della Morte E., Orioli M., Brini A.T. (2022). Lipidomics of Cell Secretome Combined with the Study of Selected Bioactive Lipids in an In Vitro Model of Osteoarthritis. Stem Cells Transl. Med..

[38] Casati S., Giannasi C., Niada S., Bergamaschi R.F., Orioli M., Brini A.T. (2021). Bioactive Lipids in MSCs Biology: State of the Art and Role in Inflammation. Int. J. Mol. Sci..

[39] Gnecchi M., Zhang Z., Ni A., Dzau V.J. (2008). Paracrine mechanisms in adult stem cell signaling and therapy. Circ. Res..

[40] Kraskiewicz H., Hinc P., Krawczenko A., Bielawska-Pohl A., Paprocka M., Witkowska D., Mohd Isa I.L., Pandit A., Klimczak A. (2021). HATMSC Secreted Factors in the Hydrogel as a Potential Treatment for Chronic Wounds-In Vitro Study. Int. J. Mol. Sci..

[41] Shin S., Lee J., Kwon Y., Park K.S., Jeong J.H., Choi S.J., Bang S.I., Chang J.W., Lee C. (2021). Comparative Proteomic Analysis of the Mesenchymal Stem Cells Secretome from Adipose, Bone Marrow, Placenta and Wharton’s Jelly. Int. J. Mol. Sci..

[42] Szyposzynska A., Bielawska-Pohl A., Krawczenko A., Doszyn O., Paprocka M., Klimczak A. (2020). Suppression of Ovarian Cancer Cell Growth by AT-MSC Microvesicles. Int. J. Mol. Sci..

[43] Krawczenko A., Bielawska-Pohl A., Paprocka M., Kraskiewicz H., Szyposzynska A., Wojdat E., Klimczak A. (2020). Microvesicles from Human Immortalized Cell Lines of Endothelial Progenitor Cells and Mesenchymal Stem/Stromal Cells of Adipose Tissue Origin as Carriers of Bioactive Factors Facilitating Angiogenesis. Stem Cells Int..

[44] Szyposzynska A., Bielawska-Pohl A., Murawski M., Sozanski R., Chodaczek G., Klimczak A. (2023). Mesenchymal Stem Cell Microvesicles from Adipose Tissue: Unraveling Their Impact on Primary Ovarian Cancer Cells and Their Therapeutic Opportunities. Int. J. Mol. Sci..

[45] Li S., Zhang J., Liu X., Wang N., Sun L., Liu J., Liu X., Masoudi A., Wang H., Li C. (2024). Proteomic characterization of hUC-MSC extracellular vesicles and evaluation of its therapeutic potential to treat Alzheimer’s disease. Sci. Rep..

[46] Zhao Y., Xiong W., Li C., Zhao R., Lu H., Song S., Zhou Y., Hu Y., Shi B., Ge J. (2023). Hypoxia-induced signaling in the cardiovascular system: Pathogenesis and therapeutic targets. Signal Transduct. Target. Ther..

[47] Zhang C.S., Shao K., Liu C.W., Li C.J., Yu B.T. (2019). Hypoxic preconditioning BMSCs-exosomes inhibit cardiomyocyte apoptosis after acute myocardial infarction by upregulating microRNA-24. Eur. Rev. Med. Pharmacol. Sci..

[48] Shao L., Chen Y., Li J., Chao J., Yang Z., Ding Y., Shen H., Chen Y., Shen Z. (2023). Hypoxia-Elicited Mesenchymal Stem Cell-Derived Small Extracellular Vesicles Alleviate Myocardial Infarction by Promoting Angiogenesis through the miR-214/Sufu Pathway. Stem Cells Int..

[49] Joshi J.M., Verma S., Upadhya R., Bhat S., Seetharam R.N. (2025). Inflammatory priming of mesenchymal stromal cells enhances its secretome potential through secretion of anti-inflammatory and ECM modulating factors: Insights into proteomic and functional properties. Biochem. Biophys. Res. Commun..

[50] Yu Y., Yoo S.M., Park H.H., Baek S.Y., Kim Y.J., Lee S., Kim Y.L., Seo K.W., Kang K.S. (2019). Preconditioning with interleukin-1 beta and interferon-gamma enhances the efficacy of human umbilical cord blood-derived mesenchymal stem cells-based therapy via enhancing prostaglandin E2 secretion and indoleamine 2,3-dioxygenase activity in dextran sulfate sodium-induced colitis. J. Tissue Eng. Regen. Med..

[51] Redondo-Castro E., Cunningham C., Miller J., Martuscelli L., Aoulad-Ali S., Rothwell N.J., Kielty C.M., Allan S.M., Pinteaux E. (2017). Interleukin-1 primes human mesenchymal stem cells towards an anti-inflammatory and pro-trophic phenotype in vitro. Stem Cell Res. Ther..

[52] Jung M., Ma Y., Iyer R.P., DeLeon-Pennell K.Y., Yabluchanskiy A., Garrett M.R., Lindsey M.L. (2017). IL-10 improves cardiac remodeling after myocardial infarction by stimulating M2 macrophage polarization and fibroblast activation. Basic. Res. Cardiol..

[53] Xu R., Zhang F., Chai R., Zhou W., Hu M., Liu B., Chen X., Liu M., Xu Q., Liu N. (2019). Exosomes derived from pro-inflammatory bone marrow-derived mesenchymal stem cells reduce inflammation and myocardial injury via mediating macrophage polarization. J. Cell Mol. Med..

[54] Matta A., Nader V., Lebrin M., Gross F., Prats A.C., Cussac D., Galinier M., Roncalli J. (2022). Pre-Conditioning Methods and Novel Approaches with Mesenchymal Stem Cells Therapy in Cardiovascular Disease. Cells.

[55] Huang P., Wang L., Li Q., Tian X., Xu J., Xu J., Xiong Y., Chen G., Qian H., Jin C. (2020). Atorvastatin enhances the therapeutic efficacy of mesenchymal stem cells-derived exosomes in acute myocardial infarction via up-regulating long non-coding RNA H19. Cardiovasc. Res..

[56] Ning Y., Huang P., Chen G., Xiong Y., Gong Z., Wu C., Xu J., Jiang W., Li X., Tang R. (2023). Atorvastatin-pretreated mesenchymal stem cell-derived extracellular vesicles promote cardiac repair after myocardial infarction via shifting macrophage polarization by targeting microRNA-139-3p/Stat1 pathway. BMC Med..

[57] Wang Q., Zhang L., Sun Z., Chi B., Zou A., Mao L., Xiong X., Jiang J., Sun L., Zhu W. (2021). HIF-1alpha overexpression in mesenchymal stem cell-derived exosome-encapsulated arginine-glycine-aspartate (RGD) hydrogels boost therapeutic efficacy of cardiac repair after myocardial infarction. Mater. Today Bio.

[58] Kang K., Ma R., Cai W., Huang W., Paul C., Liang J., Wang Y., Zhao T., Kim H.W., Xu M. (2015). Exosomes Secreted from CXCR4 Overexpressing Mesenchymal Stem Cells Promote Cardioprotection via Akt Signaling Pathway following Myocardial Infarction. Stem Cells Int..

[59] Paprocka M., Kraskiewicz H., Bielawska-Pohl A., Krawczenko A., Maslowski L., Czyzewska-Buczynska A., Witkiewicz W., Dus D., Czarnecka A. (2021). From Primary MSC Culture of Adipose Tissue to Immortalized Cell Line Producing Cytokines for Potential Use in Regenerative Medicine Therapy or Immunotherapy. Int. J. Mol. Sci..

[60] Kim Y.H., Lee H.J., Cho K.A., Woo S.Y., Ryu K.H. (2022). Conditioned medium from human tonsil-derived mesenchymal stem cells inhibits glucocorticoid-induced adipocyte differentiation. PLoS ONE.

[61] Shao L., Zhang Y., Lan B., Wang J., Zhang Z., Zhang L., Xiao P., Meng Q., Geng Y.J., Yu X.Y. (2017). MiRNA-Sequence Indicates That Mesenchymal Stem Cells and Exosomes Have Similar Mechanism to Enhance Cardiac Repair. Biomed. Res. Int..

[62] He J.G., Li H.R., Han J.X., Li B.B., Yan D., Li H.Y., Wang P., Luo Y. (2018). GATA-4-expressing mouse bone marrow mesenchymal stem cells improve cardiac function after myocardial infarction via secreted exosomes. Sci. Rep..

[63] Alrefai M.T., Tarola C.L., Raagas R., Ridwan K., Shalal M., Lomis N., Paul A., Alrefai M.D., Prakash S., Schwertani A. (2019). Functional Assessment of Pluripotent and Mesenchymal Stem Cell Derived Secretome in Heart Disease. Ann. Stem Cell Res..

[64] Ma J., Zhao Y., Sun L., Sun X., Zhao X., Sun X., Qian H., Xu W., Zhu W. (2017). Exosomes Derived from Akt-Modified Human Umbilical Cord Mesenchymal Stem Cells Improve Cardiac Regeneration and Promote Angiogenesis via Activating Platelet-Derived Growth Factor D. Stem Cells Transl. Med..

[65] Ni J., Liu X., Yin Y., Zhang P., Xu Y.W., Liu Z. (2019). Exosomes Derived from TIMP2-Modified Human Umbilical Cord Mesenchymal Stem Cells Enhance the Repair Effect in Rat Model with Myocardial Infarction Possibly by the Akt/Sfrp2 Pathway. Oxid. Med. Cell Longev..

[66] Jin H.J., Bae Y.K., Kim M., Kwon S.J., Jeon H.B., Choi S.J., Kim S.W., Yang Y.S., Oh W., Chang J.W. (2013). Comparative analysis of human mesenchymal stem cells from bone marrow, adipose tissue, and umbilical cord blood as sources of cell therapy. Int. J. Mol. Sci..

[67] Romanov Y.A., Volgina N.E., Vtorushina V.V., Romanov A.Y., Dugina T.N., Kabaeva N.V., Sukhikh G.T. (2019). Comparative Analysis of Secretome of Human Umbilical Cord- and Bone Marrow-Derived Multipotent Mesenchymal Stromal Cells. Bull. Exp. Biol. Med..

[68] Mathew B., Ravindran S., Liu X., Torres L., Chennakesavalu M., Huang C.C., Feng L., Zelka R., Lopez J., Sharma M. (2019). Mesenchymal stem cell-derived extracellular vesicles and retinal ischemia-reperfusion. Biomaterials.

[69] Liu C., Wang C., Yang F., Lu Y., Du P., Hu K., Yin X., Zhao P., Lu G. (2022). The conditioned medium from mesenchymal stromal cells pretreated with proinflammatory cytokines promote fibroblasts migration and activation. PLoS ONE.

[70] Acuto S., Lo Iacono M., Baiamonte E., Lo Re R., Maggio A., Cavalieri V. (2023). An optimized procedure for preparation of conditioned medium from Wharton’s jelly mesenchymal stromal cells isolated from umbilical cord. Front. Mol. Biosci..

[71] Ranganath S.H., Levy O., Inamdar M.S., Karp J.M. (2012). Harnessing the mesenchymal stem cell secretome for the treatment of cardiovascular disease. Cell Stem Cell.

[72] An C., Zhao Y., Guo L., Zhang Z., Yan C., Zhang S., Zhang Y., Shao F., Qi Y., Wang X. (2025). Innovative approaches to boost mesenchymal stem cells efficacy in myocardial infarction therapy. Mater. Today Bio.

[73] Sid-Otmane C., Perrault L.P., Ly H.Q. (2020). Mesenchymal stem cell mediates cardiac repair through autocrine, paracrine and endocrine axes. J. Transl. Med..

[74] Alijani-Ghazyani Z., Roushandeh A.M., Sabzevari R., Salari A., Razavi Toosi M.T., Jahanian-Najafabadi A., Roudkenar M.H. (2021). Conditioned medium harvested from Hif1alpha engineered mesenchymal stem cells ameliorates LAD-occlusion -induced injury in rat acute myocardial ischemia model. Int. J. Biochem. Cell Biol..

[75] Qin D., Wang X., Pu J., Hu H. (2024). Cardiac cells and mesenchymal stem cells derived extracellular vesicles: A potential therapeutic strategy for myocardial infarction. Front. Cardiovasc. Med..

[76] Xu C.M., Sabe S.A., Brinck-Teixeira R., Sabra M., Sellke F.W., Abid M.R. (2023). Visualization of cardiac uptake of bone marrow mesenchymal stem cell-derived extracellular vesicles after intramyocardial or intravenous injection in murine myocardial infarction. Physiol. Rep..

[77] Kastner N., Mester-Tonczar J., Winkler J., Traxler D., Spannbauer A., Ruger B.M., Goliasch G., Pavo N., Gyongyosi M., Zlabinger K. (2020). Comparative Effect of MSC Secretome to MSC Co-culture on Cardiomyocyte Gene Expression Under Hypoxic Conditions in vitro. Front. Bioeng. Biotechnol..

[78] Angoulvant D., Ivanes F., Ferrera R., Matthews P.G., Nataf S., Ovize M. (2011). Mesenchymal stem cell conditioned media attenuates in vitro and ex vivo myocardial reperfusion injury. J. Heart Lung Transpl..

[79] Lee T.L., Lai T.C., Lin S.R., Lin S.W., Chen Y.C., Pu C.M., Lee I.T., Tsai J.S., Lee C.W., Chen Y.L. (2021). Conditioned medium from adipose-derived stem cells attenuates ischemia/reperfusion-induced cardiac injury through the microRNA-221/222/PUMA/ETS-1 pathway. Theranostics.

[80] Charles C.J., Li R.R., Yeung T., Mazlan S.M.I., Lai R.C., de Kleijn D.P.V., Lim S.K., Richards A.M. (2020). Systemic Mesenchymal Stem Cell-Derived Exosomes Reduce Myocardial Infarct Size: Characterization With MRI in a Porcine Model. Front. Cardiovasc. Med..

[81] Lv K., Li Q., Zhang L., Wang Y., Zhong Z., Zhao J., Lin X., Wang J., Zhu K., Xiao C. (2019). Incorporation of small extracellular vesicles in sodium alginate hydrogel as a novel therapeutic strategy for myocardial infarction. Theranostics.

[82] Tang J., Jin L., Liu Y., Li L., Ma Y., Lu L., Ma J., Ding P., Yang X., Liu J. (2020). Exosomes Derived from Mesenchymal Stem Cells Protect the Myocardium Against Ischemia/Reperfusion Injury Through Inhibiting Pyroptosis. Drug Des. Devel Ther..

[83] Dong F., Patnaik S., Duan Z.H., Kiedrowski M., Penn M.S., Mayorga M.E. (2017). A Novel Role for CAMKK1 in the Regulation of the Mesenchymal Stem Cell Secretome. Stem Cells Transl. Med..

[84] Ju C., Li Y., Shen Y., Liu Y., Cai J., Liu N., Ma G., Tang Y. (2018). Transplantation of Cardiac Mesenchymal Stem Cell-Derived Exosomes for Angiogenesis. J. Cardiovasc. Transl. Res..

[85] Luther K.M., Haar L., McGuinness M., Wang Y., Lynch Iv T.L., Phan A., Song Y., Shen Z., Gardner G., Kuffel G. (2018). Exosomal miR-21a-5p mediates cardioprotection by mesenchymal stem cells. J. Mol. Cell Cardiol..

[86] Wang X., Chen Y., Zhao Z., Meng Q., Yu Y., Sun J., Yang Z., Chen Y., Li J., Ma T. (2018). Engineered Exosomes With Ischemic Myocardium-Targeting Peptide for Targeted Therapy in Myocardial Infarction. J. Am. Heart Assoc..

[87] Ma T., Chen Y., Chen Y., Meng Q., Sun J., Shao L., Yu Y., Huang H., Hu Y., Yang Z. (2018). MicroRNA-132, Delivered by Mesenchymal Stem Cell-Derived Exosomes, Promote Angiogenesis in Myocardial Infarction. Stem Cells Int..

[88] Mao Q., Liang X.L., Zhang C.L., Pang Y.H., Lu Y.X. (2019). LncRNA KLF3-AS1 in human mesenchymal stem cell-derived exosomes ameliorates pyroptosis of cardiomyocytes and myocardial infarction through miR-138-5p/Sirt1 axis. Stem Cell Res. Ther..

[89] Zhao J., Li X., Hu J., Chen F., Qiao S., Sun X., Gao L., Xie J., Xu B. (2019). Mesenchymal stromal cell-derived exosomes attenuate myocardial ischaemia-reperfusion injury through miR-182-regulated macrophage polarization. Cardiovasc. Res..

[90] Zheng H., Liang X., Han Q., Shao Z., Zhang Y., Shi L., Hong Y., Li W., Mai C., Mo Q. (2021). Hemin enhances the cardioprotective effects of mesenchymal stem cell-derived exosomes against infarction via amelioration of cardiomyocyte senescence. J. Nanobiotechnol..

[91] Zhang N., Song Y., Huang Z., Chen J., Tan H., Yang H., Fan M., Li Q., Wang Q., Gao J. (2020). Monocyte mimics improve mesenchymal stem cell-derived extracellular vesicle homing in a mouse MI/RI model. Biomaterials.

[92] Yao J., Huang K., Zhu D., Chen T., Jiang Y., Zhang J., Mi L., Xuan H., Hu S., Li J. (2021). A Minimally Invasive Exosome Spray Repairs Heart after Myocardial Infarction. ACS Nano.

